# Supplementary material for: Citrin mediated metabolic rewiring in response to altered basal subcellular Ca2+ homeostasis
Source: Commun Biol. 2022 Jan 20;5:76. doi: 10.1038/s42003-022-03019-2 (PMC8776887; doi:10.1038/s42003-022-03019-2)
Supplement: Supplementary file 2 — Supplementary Figures [file 42003_2022_3019_MOESM2_ESM.pdf]

Supplementary figures.

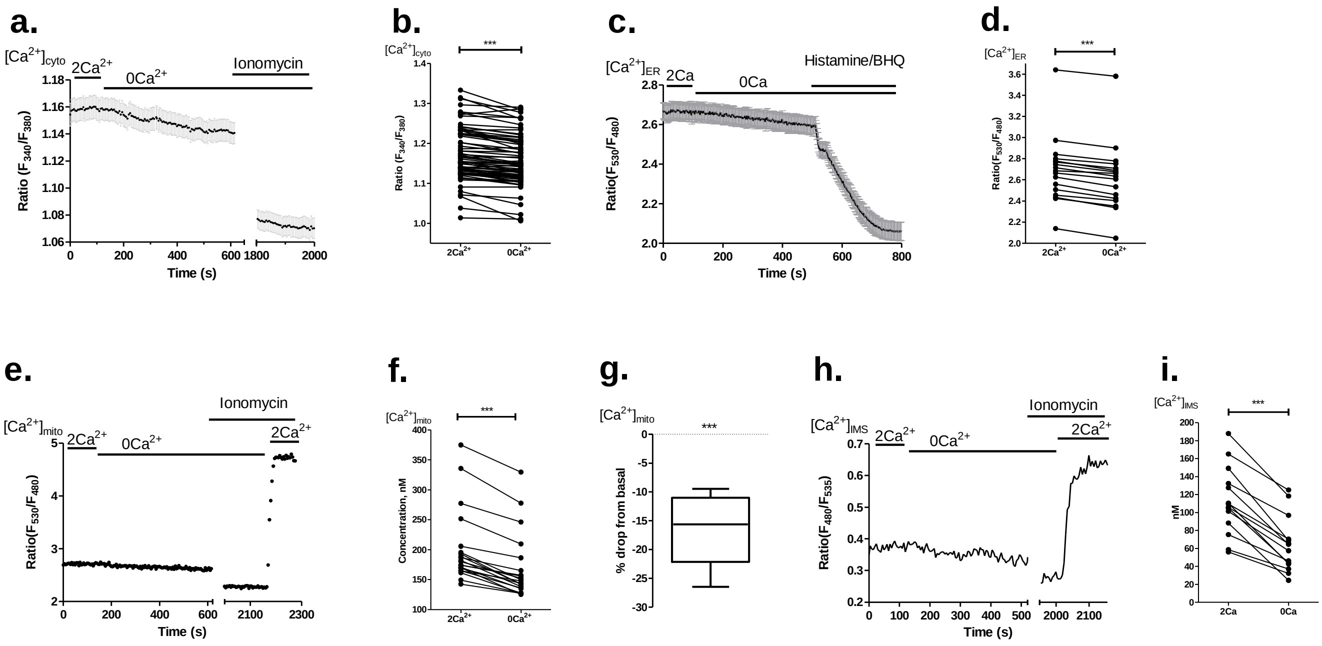

Supplementary figure 1. Protocols and statistics for Ca^{2+} measurements in cytosol (**a-b)**, ER **(c-d)**, mitochondria **(e-g)** and IMS **(h-i)**, average traces ±SEM are shown; **b.** Paired analysis of cytosolic Ca^{2+} levels before and after 6 minutes of extracellular Ca^{2+} removal (paired t-test, ***p<0.0001, n=3, 68 cells). **d.** Same as in **b**, but in ER (paired t-test, ***p<0.0001, n=9, 17 cells). **f.** Paired analysis of mitochondrial Ca^{2+} concentration before and after 6 minutes of extracellular Ca^{2+} removal (paired t-test, ***p<0.0001, n=4, 21 cells). **g.** Percentage drop of basal mitochondrial Ca^{2+} after 6 minutes perfusion with $0Ca^{2+}$ buffer (paired t-test, ***p<0.0001, n=4, 21 cells). **i.** Paired analysis of IMS Ca^{2+} concentration before and after 6 minutes of extracellular Ca^{2+} removal (paired t-test, ***p<0.0001, n=6, 13 cells).

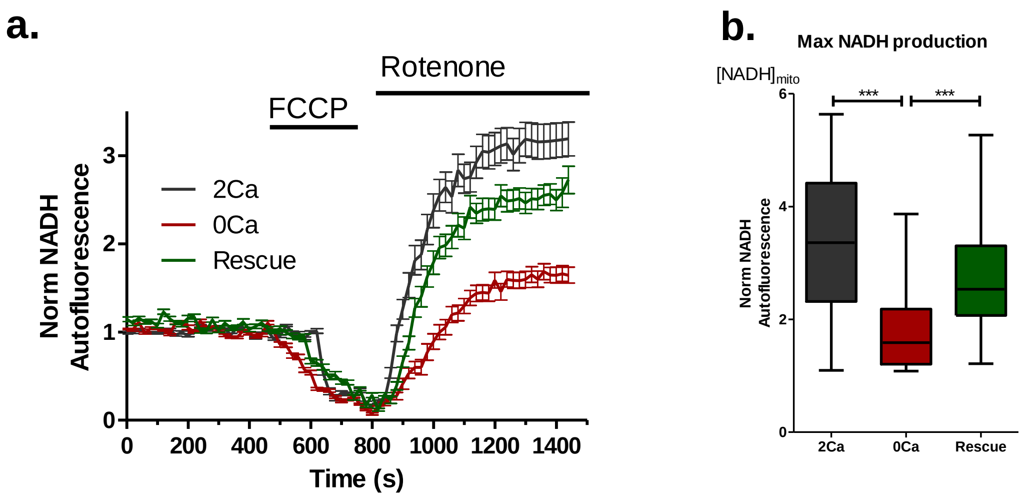

Supplementary figure 2. Ca^{2+} re-addition rescues reduced mitochondrial NADH production resulting from disrupted sub-cellular Ca^{2+} homeostasis. **a.** Average traces of mitochondrial NADH autofluorescence measurements (±SEM), rescue represents cells perfused with $0Ca^{2+}$ buffer for 6 minutes followed by $2Ca^{2+}$ buffer for 10 minutes. **b.** Statistical analysis of maximal NADH production from **a** (One-way ANOVA with Tukey's multiple comparison test, ***p<0.001, n=3-4, 50-73 cells).

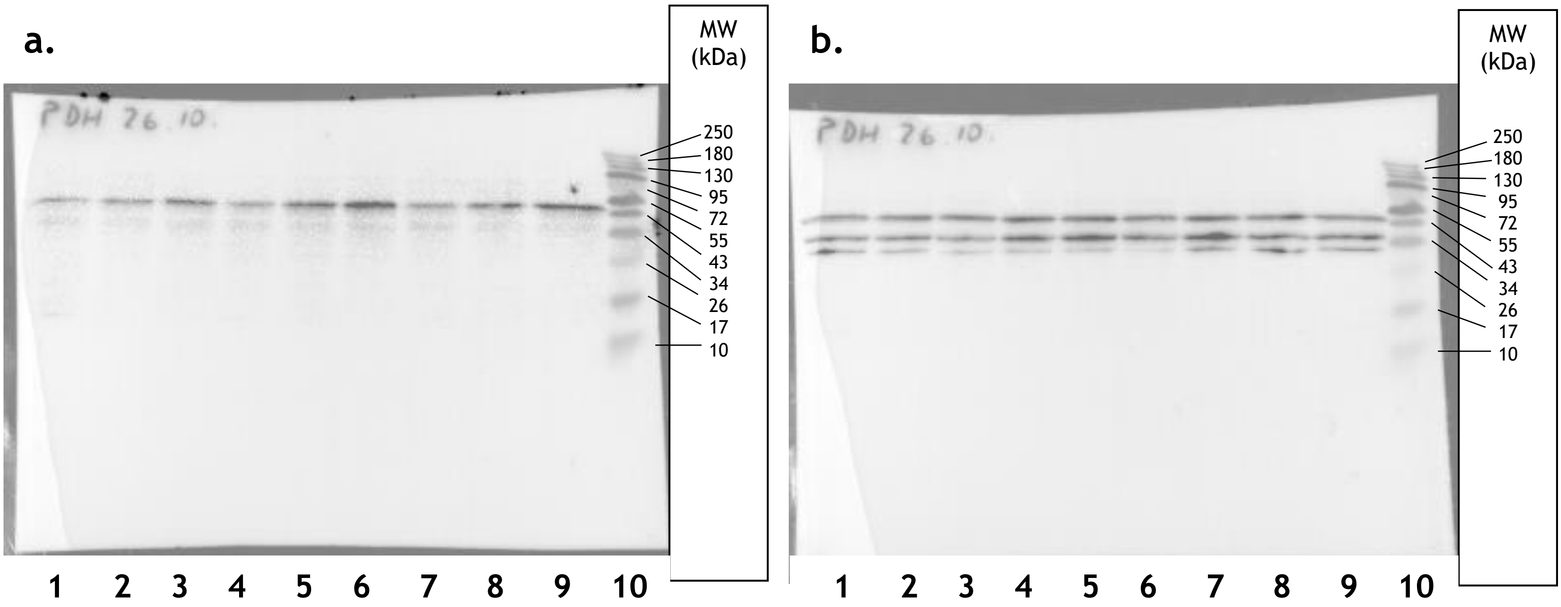

Supplementary figure 3. Unprocessed blots of phosphorylated PDH at Ser293 **(a)** and total PDH after stripping the blot shown in panel a **(b).** Lanes 1, 4 and 7 are 2Ca^{2+}, lanes 2, 5, and 8 are 5 min 0Ca^{2+}, and lanes 3, 6, and 9 are 1 h in 0Ca^{2+} conditions. Lanes 1-3, 4-6 and 7-9 are biological replicates. Lane 10 is the molecular weight (MW) ladder.

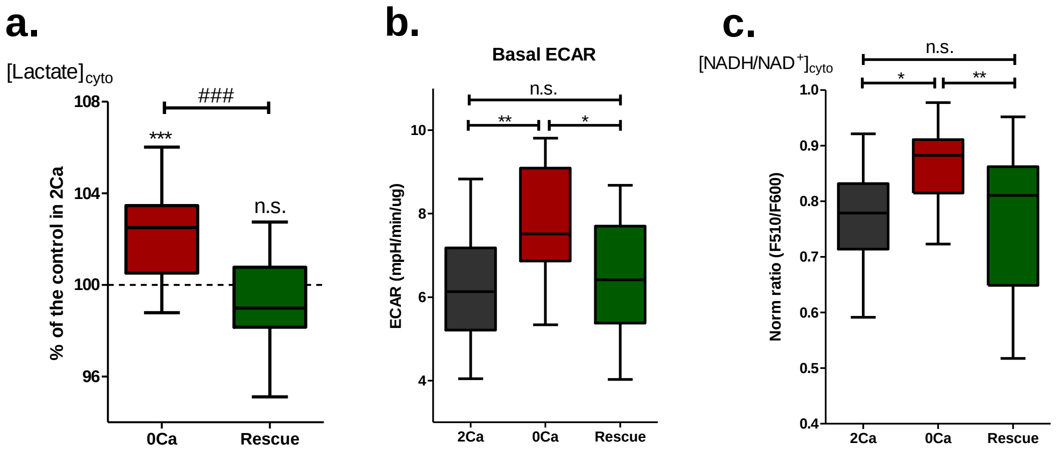

Supplementary figure 4. Ca^{2+} re-addition rescues metabolic alterations resulting from disrupted sub-cellular Ca^{2+} homeostasis. **a.** Cytosolic lactate levels after 6 minutes of Ca^{2+} removal (0Ca^{2+}) followed by 10 minutes of Ca^{2+} re-addition (rescue); the dashed line represents initial lactate level in 2Ca^{2+} buffer (Repeated Measures ANOVA with Tukey's multiple comparison test, ***p<0.001

against $2Ca^{2+}$, ###p<0.001 against rescue, n=7, 14 cells). **b.** Statistical analysis of ECAR; for the rescue, ECAR was assessed following injection of 25 µl 7x Ca^{2+} buffer after basal ECAR in $0Ca^{2+}$ was measured (One-way ANOVA with Tukey's multiple comparison test, *p<0.05, **p<0.01, n=24-27). **c.** Comparison of cytosolic NADH/NAD^+ ratio after 6 minutes in $2Ca^{2+}$, $0Ca^{2+}$ or 6 minutes in $0Ca^{2+}$ followed by 10 minutes in $2Ca^{2+}$ buffer (rescue), (One-way ANOVA with Tukey's multiple comparison test, *p<0.05, **p<0.01, n=4-6, 15-20 cells).

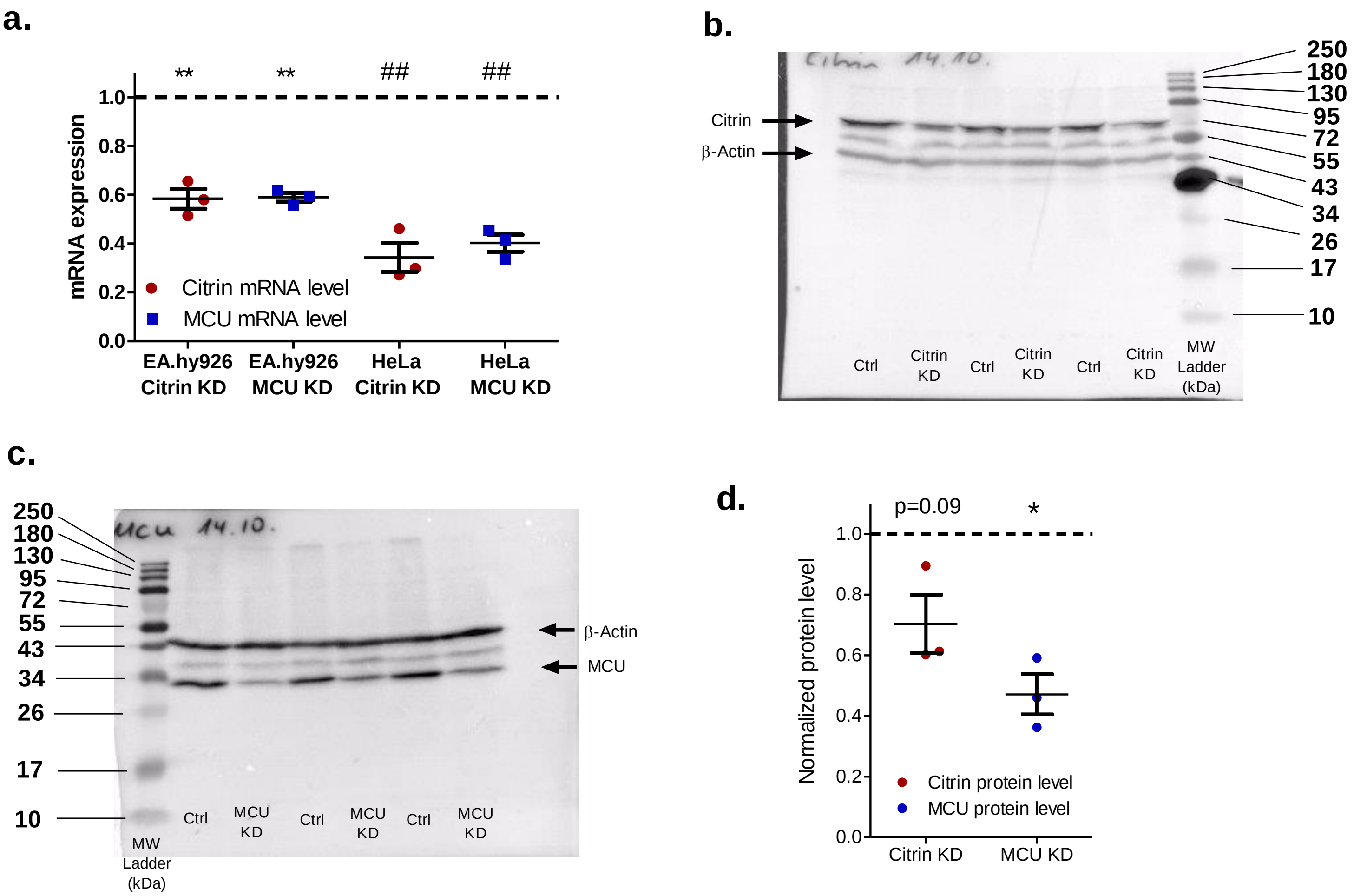

Supplementary figure 5. Citrin and MCU expression level after respective knockdowns with specific siRNAs. **a.** mRNA levels of citrin and MCU after KD in EA.hy926 and HeLa cell lines; the horizontal dotted line represents normalized mRNA level of Citrin and MCU in control groups for respective cell lines (paired t-test, n=3, EA.hy926 ** - p=0.01, HeLa ## - p<0.01). **b.** Western blot of citrin in control and citrin KD EA.hy926 cells; β-Actin was used as a loading control. **c.** Western

blot of MCU in control and citrin KD EA.hy926 cells; β-Actin was used as a loading control. **d.** Statistical analysis of **b** and **c;** the horizontal dotted line represents normalized protein level of Citrin and MCU in respective control groups (paired t-test, n=3, p=0.09 for citrin KD, *p=0.015 for MCU KD). Error bars show SEM

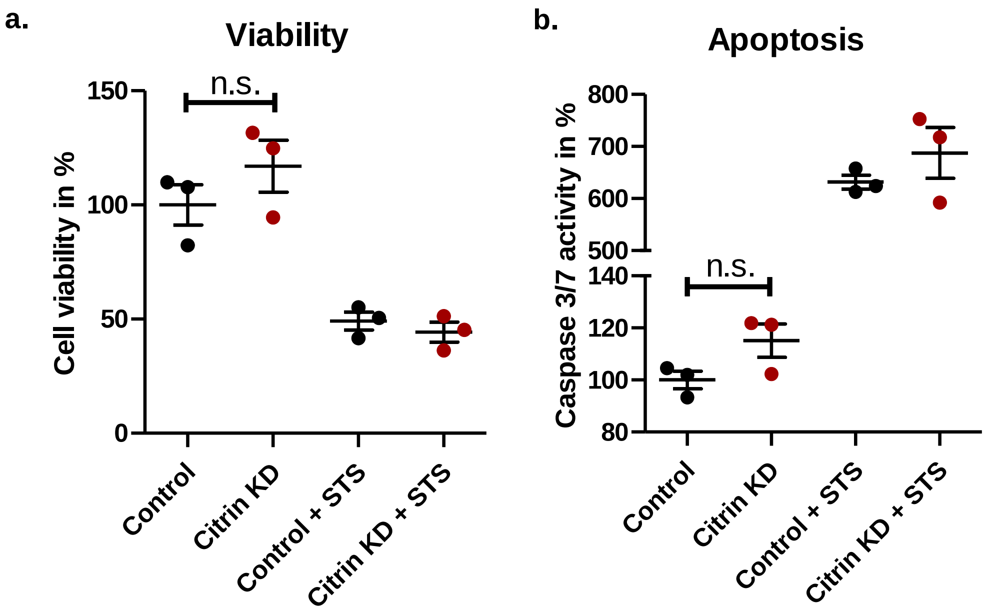

Supplementary figure 6. Citrin KD doesn't critically affect cell viability **(a.)** and apoptosis **(b.)** 48 hours post transfection; Staurosporine (STS) (unpaired t-test, n=3, n.s.for **a**, p=0.3, n.s. for **b,** **p**=0.1). Error bars show SEM.

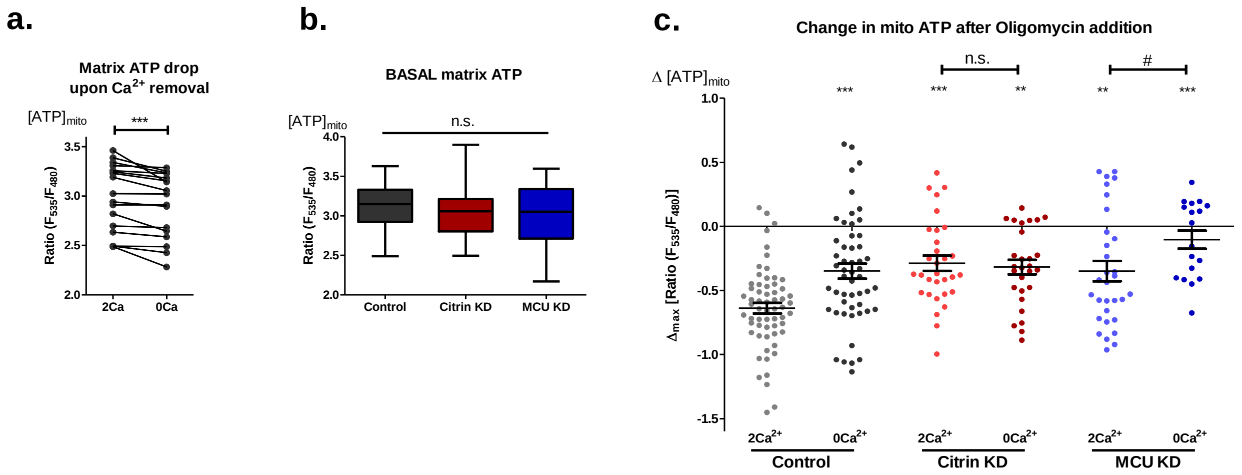

Supplementary figure 7. Mitochondrial ATP measurements in HeLa cells. **a.** Statistical analysis of the change in mitochondrial ATP level after 5 min perfusion with $0Ca^{2+}$ buffer (paired-t test, n=6, 17 cells, ***p<0.0001). **b.** Statistical analysis of basal mitochondrial ATP levels represented by mtAT1.03 ratio in control, MCU KD and Citrin KD, One-way ANOVA with Tukey's multiple comparison test, n=8-11, 33 cells (control), 37 cells (Citrin KD), 28 cells (MCU KD). **c.** Statistical analysis of the change in mitochondrial ATP level after 2µM oligomycin addition in control, MCU KD and Citrin KD cells +/- extracellular Ca^{2+} (One-way ANOVA with Tukey's Multiple Comparison Test, **p<0.01, ***p<0.001, n=5-15, 18-61 cells). Error bars show SEM.

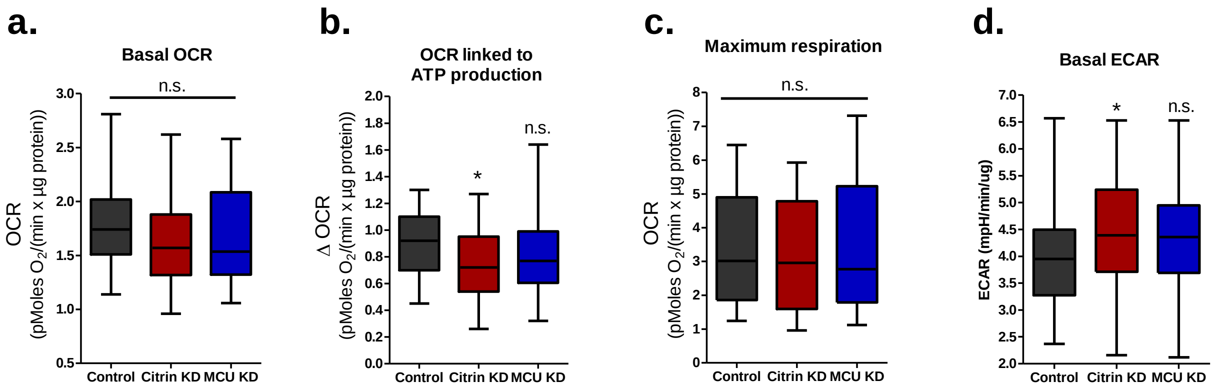

Supplementary figure 8. Statistical analysis of basal **(a.)**, ATP linked **(b.)** and maximal **(c.)** OCR and ECAR **(d.)** of control, citrin KD and MCU KD cells (One-Way ANOVA with Tukey's Multiple Comparison Test, *p<0.05, n=35-40).

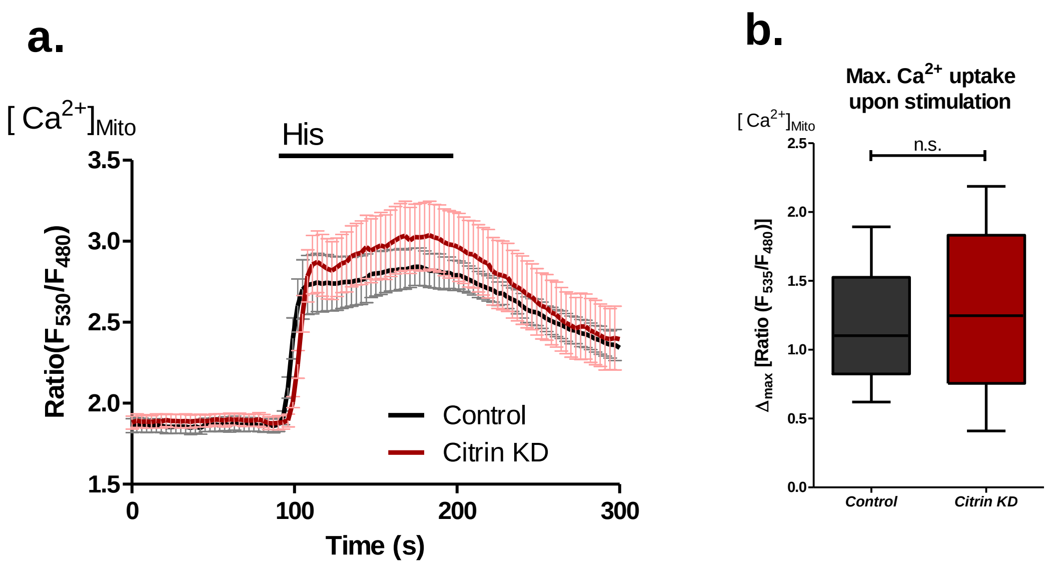

Supplementary figure 9. Citrin KD doesn't affect mitochondrial Ca^{2+} uptake upon stimulation with IP_3 generating agonist. **a.** Average traces of mitochondrial Ca^{2+} dynamics displayed as emission ratio of mitochondrial matrix targeted Ca^{2+} probe mtD3cpv (±SEM). **b.** Statistical analysis of mitochondrial Ca^{2+} uptake from **a** (unpaired t-test, n=9-12, n.s., p=0.54).

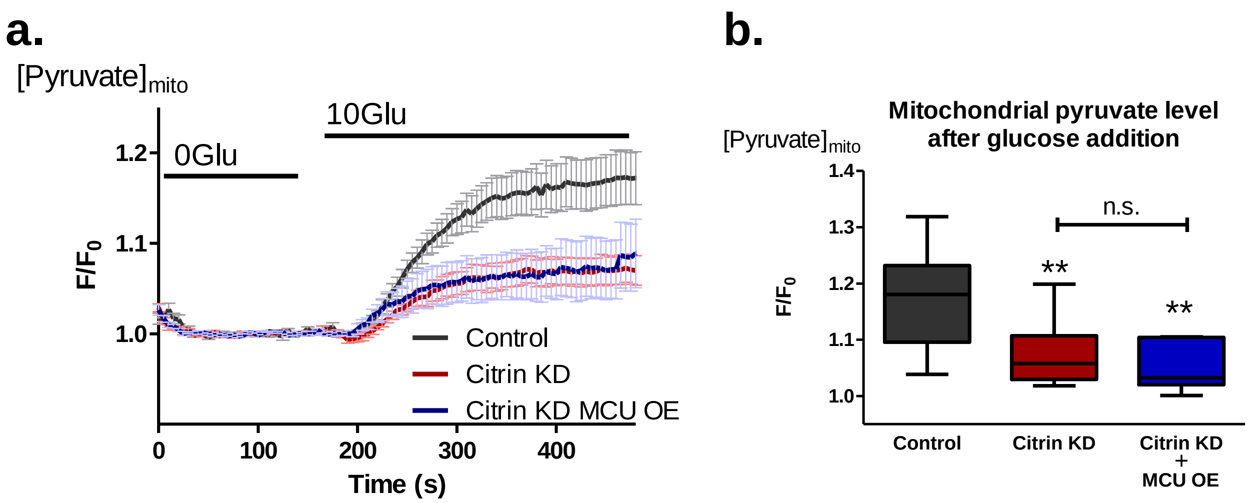

Supplementary figure 10. MCU OE doesn't rescue metabolic defects of citrin KD. **a.** Average traces of mitochondrial pyruvate measurements with mito-PyronicSF (±SEM) in the presence of extracellular Ca^{2+} under control, citrin KD and citrin KD and MCU OE conditions. **b.** Statistical analysis of **a** (One-way ANOVA with Tukey's multiple comparison test, **p<0.01, n=7-14).
