# Supplementary material for: Citrin mediated metabolic rewiring in response to altered basal subcellular Ca2+ homeostasis
Source: Commun Biol. 2022 Jan 20;5:76. doi: 10.1038/s42003-022-03019-2 (PMC8776887; doi:10.1038/s42003-022-03019-2)
Supplement: Supplementary file 3 — Description of Additional Supplementary Files [file 42003_2022_3019_MOESM3_ESM.pdf]

Description of Additional Supplementary Data Files

File name: Supplementary Data 1

Description: Source data underlying the graphs and charts presented in the main figures (Fig. 1, panels a, c, e; Fig. 2, panels a-h; Fig. 3, panels a-g, I; Fig. 4, panels a-h; Fig 5, panels a-g; Fig. 6, panels a-f; Fig. 7, panels a-f; Fig. 8, panels a-e) and Supplementary figures (Supplementary fig. 1, panels a-i; Supplementary fig. 2, panels a and b; Supplementary fig. 4, panels a-c; Supplementary fig. 5, panels a and d; Supplementary fig. 6, panels a and b; Supplementary fig. 7, panels a-c; Supplementary fig. 8, panels a-d; Supplementary fig. 9, panels a and b; Supplementary fig. 10, panels a and b).
